# Patellar malalignment: A common disorder associated with knee pain

**DOI:** 10.1016/j.bj.2023.100658

**Published:** 2023-09-05

**Authors:** Chi-Chuan Wu

**Affiliations:** Department of Orthopedic Surgery, Chang Gung Memorial Hospital, Chang Gung University, Taoyuan, Taiwan

**Keywords:** Knee joint, Malalignment, Pain, Patellofemoral, Tibiofemoral

## Abstract

Pain-associated knee joint disorders are common in daily life. Practically, knee pain should be divided into the origin from the isolated tibiofemoral (TF), isolated patellofemoral (PF) joint, or a combination thereof. The TF joint controls the actions of level walking, while the PF joint controls knee flexion-extension. Owing to its sufficient inherent stability, non-traumatic disorders of the isolated TF joint in young individuals are uncommon. In contrast, because of its insufficient inherent stability, non-traumatic disorders of the isolated PF joint are common in young individuals. Patellar malalignment (PM) associated with knee pain is common in all age groups, and the most common predisposing factor is imbalanced peripatellar soft-tissue tension. The outward forces acting on the patella are caused by pulling from the quadriceps femoris during knee flexion to extension (manifested by the quadriceps angle [Q-angle]), and sliding backward of the iliotibial band (ITB) during knee extension to flexion. Once the muscle power of the vastus medialis (especially the vastus medialis obliquus [VMO]) decreases, which lowers the counteracting effect against outward forces, the patella displaces or rotates laterally. The reduced contact surface between the patella and the femoral condyle significantly increases the compressive pressure and injures the articular cartilage. Subsequently, progressive PF degeneration occurs. Although other factors may also cause PM, they are relatively uncommon. In principle, nonsurgical treatment of PM should be considered first, while surgical treatment should follow established indications. Some nonsurgical techniques are currently widely used that feature high satisfaction rates. Surgical techniques are continuously being developed, and their success rates have gradually improved. This study aimed to review the current literature for relevant studies and report related publications of the author's institution to emphasize the universality and importance of PM management. Conceptually, simply focusing on problems of the TF joint cannot treat all knee disorders.

## Introduction

Knee joint disorders are common and include traumatic or non-traumatic origins. Anatomically, the knee joint consists of the tibiofemoral (TF) and patellofemoral (PF) joints, each of which performs different actions. The TF joint controls movement during level walking, while the PF joint is involved in knee flexion-extension. The range of motion of the TF joint during level walking is 0-60° [1,2]. During stair climbing (usually >80°), the PF joint is proportionally involved [1,3].

Because of the sufficient inherent stability of the TF joint (provided by bone shape, ligaments, and antagonistic muscles), non-traumatic disorders are uncommon in young individuals. Insidious and progressive degeneration generally occurs after 53 years of age [4]. In contrast, the PF joint is inherently unstable. The patella moves along the trochlear groove, which is shallow and wide, in an up-down direction. Additionally, the quadriceps femoris and patellar tendon pull the patella laterally during knee extension [5]. Therefore, patellar malalignment (PM) is common [6].

Clinically, symptomatic isolated PF joints cause fewer complaints than isolated TF joints [7]. A symptomatic TF joint always disturbs patients’ daily lives during walking. Treatment is generally requested as soon as possible. However, the symptoms of an isolated PF joint can be largely tolerated as long as the modes of action are modified [8]. Techniques for the treatment of TF joints with nonsurgical or surgical approaches have been developed, and brilliant achievements have been achieved. In contrast, the treatment of symptomatic isolated PF joints has resulted in a few concerns. Thus, consensus is lacking on the etiology and pathognomonic mechanisms of non-traumatic PF disorders. Therefore, optimal techniques for treatment of PM require further development [6].

With the advancement of human civilization and modern medicine, knee flexion and extension are commonly requested (for exercise or working), and improved techniques must be continuously developed. The objective of this study aimed to clarify the deviated concept about knee pain in the normal population. In fact, the vast majority of patients with PF pain can be effectively treated with rehabilitation techniques without medication or surgery. This study uses an article review mode. The author elaborately reviews the current literature for relevant studies. The normal and pathognomonic PF biomechanics are analyzed. The most reasonable steps to prevent and treat PM are clarified. Consequently, treatment of knee pain will become more efficient.

## Patellar anatomy and biomechanics

**Fig. 1:**
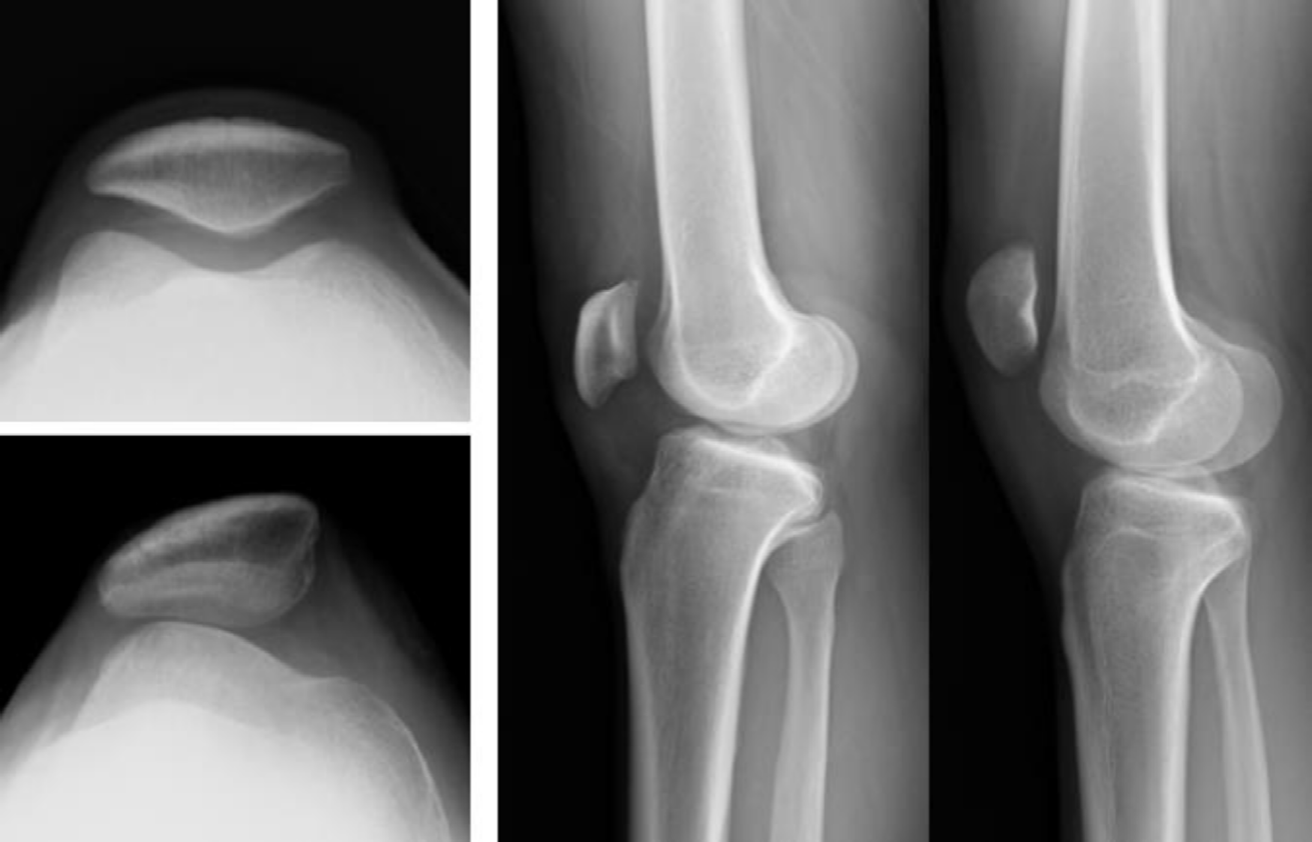
Comparison of normal and abnormal features on radiographs: normal (left upper) or dysplastic trochlear groove (left lower); normal location (middle) or high-riding (patella alta) of the patella (right).

The patella is located at the front of the knee joint. However, its inherent location has not been reported in the literature. Based on numerous classic anatomic textbooks, with full knee extension, the upper pole of the patella should be at the level of the transcondylar line of the femur and the lower pole is at the TF joint line (Fig. 1) [3,9]. A short quadriceps tendon pulls the patella proximally in association with the patella alta [10]. A short patellar tendon pulls the patella distally in association with the patella baja [11]. Clinically, patella alta can lead to PM, while the patella baja can interfere with knee flexion [11,12]. The normal range of motion of the knee is 0-140° [1,3,13].

The patellar function mainly promotes leverage efficiency in knee extension, which upgrades the moment of resistance [1,3]. Without the patella, the quadriceps tendon will slide directly on the trochlear groove. Consequently, the quadriceps tendon is vulnerable. In patients with severely comminuted patellar fractures, the patella is completely excised (by total patellectomy), which severely hinders knee extension [14].

Biomechanically, the patella sustains loads of 3–5 times the body's weight during level walking [1]. During running or jumping, loads are massively elevated, reaching as high as 10 times the body's weight [15]. Biologically, the articular cartilage is highly sensitive to applied pressure loading [16]. When PM occurs, the contact surface between the patella and the femoral condyle is significantly reduced, whereas the contact pressure greatly increases. In the early stage, the chondrocytes of the patella and the femoral condyle sustain injury. If the damage continues, the articular cartilage and subchondral tissues become massively worn. Therefore, progressive degeneration is unavoidable [16]. Effective correction of the PM is the most reasonable principle for preventing deterioration of the PF joint. However, despite numerous PM treatment techniques, consensus is lacking on the optimal technique [6].

## Pathognomonic mechanism of patellar malalignment

The causes of PM have been enthusiastically pursued, and three predisposing factors have been advocated: imbalanced peripatellar soft-tissue tension, lower-extremity malalignment, and bone or muscle anomalies (Table 1) [17]. Among them, the first factor is considered the most important, for which nonsurgical treatment is generally effective.

**Table 1:** Etiologies to cause patellar malalignment with common clinical manifestations.

Causes	Disorders
Imbalanced peripatellar	VMO hypoplasia, large Q-angle, tight ITB (snapping hip)
soft-tissue tension	
Lower extremity malalignment	Deformed valgus knee, internally torsional femur, externally torsional tibia, externally verted foot
Bone or muscle anomalies	Trochlear dysplasia, patella alta

Abbreviations: ITB: iliotibial band; Q-angle: quadriceps angle; VMO: vastus medialis obliquus.

Anatomically, the quadriceps femoris, patella, and patellar tendon form a medial angulation (i.e., the quadriceps angle [Q-angle]) [16]. When the quadriceps femoris contracts, the outward component forces cause lateral patellar displacement. In the normal patellar position, the outward forces provided by the lateral soft-tissues are counteracted by forces of the vastus medialis (especially the vastus medialis obliquus [VMO]). Once the VMO's power decreases, PM occurs [18].

In contrast, when the knee is flexed, the iliotibial band (ITB) is placed under tension. Because the ITB cannot be lengthened (owing to its fascia consistency), it is forced to slide backward [19]. Subsequently, the patella is pulled outward. To maintain normal patellar position, the VMO must play a counteractive role. In other words, the VMO must be functional at any time during knee flexion-extension to prevent PM [20].

Individuals with lower extremity malalignment (e.g., a deformed genu valgum, internally torsional femur, externally torsional tibia, or externally verted foot) have larger outward pulling forces on the patella (increased Q-angle) [21,22]. For some patients in whom physical therapy is ineffective, surgical correction of bone alignment is necessary to prevent progressive deterioration of the PF joint [23].

Individuals with bone or muscle anomalies (e.g., trochlear dysplasia or patella alta) have a very unstable patella for which nonsurgical treatment is generally ineffective (Fig. 1) [24,25]. Therefore, surgical intervention is necessary in such cases. However, despite the development of surgical techniques, outcomes remain unpredictable and require continuous development [26].

## Pathologies caused by patellar malalignment

**Fig. 2:**
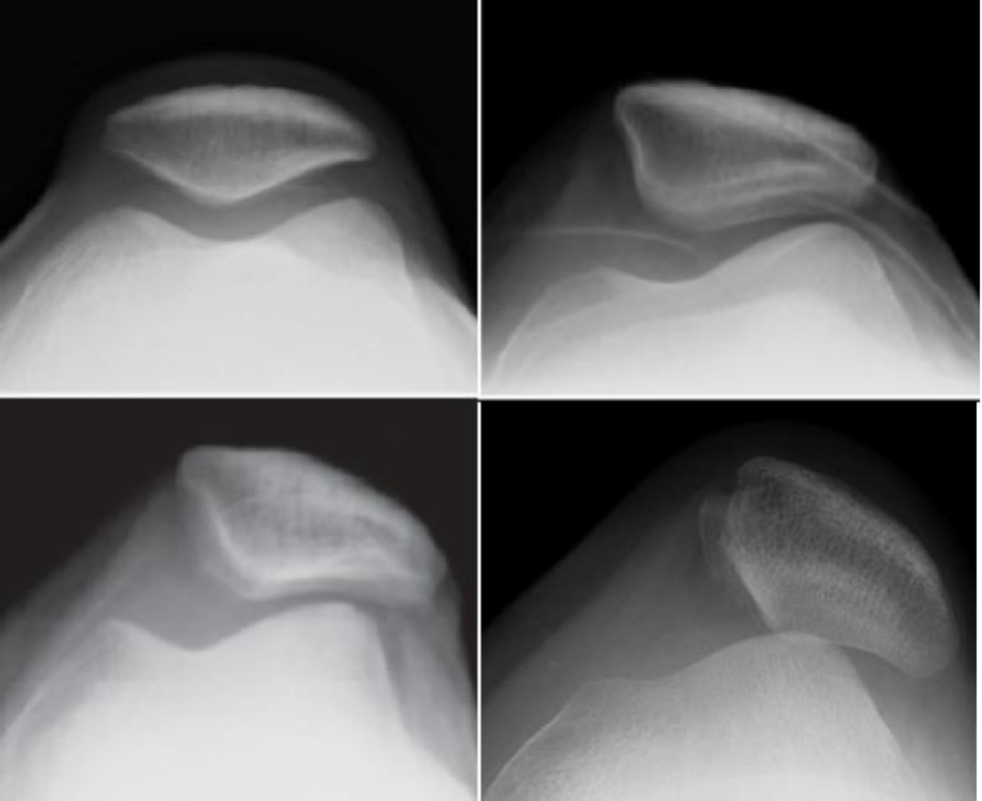
Varied types of patellar malalignment: normal (left upper), tilted (right upper), subluxed (left lower) or dislocated (right lower). The difference is based on the relative position of the patellar ridge toward the lateral femoral condyle.

Clinically and radiologically, PM can be divided into three distinct types: tilted, subluxed or dislocated [27]. This distinction is based on the relative position of the patellar ridge toward the lateral femoral condyle on axial-view radiographs (Fig. 2). In the tilted type, the patellar ridge points to the bottom of the trochlear groove. In the subluxed type, the patellar ridge is displaced outward and is located between the bottom of the trochlear groove and the condylar edge. In the dislocated type, the patellar ridge is displaced to adjoin the condylar edge.

**Fig. 3:**
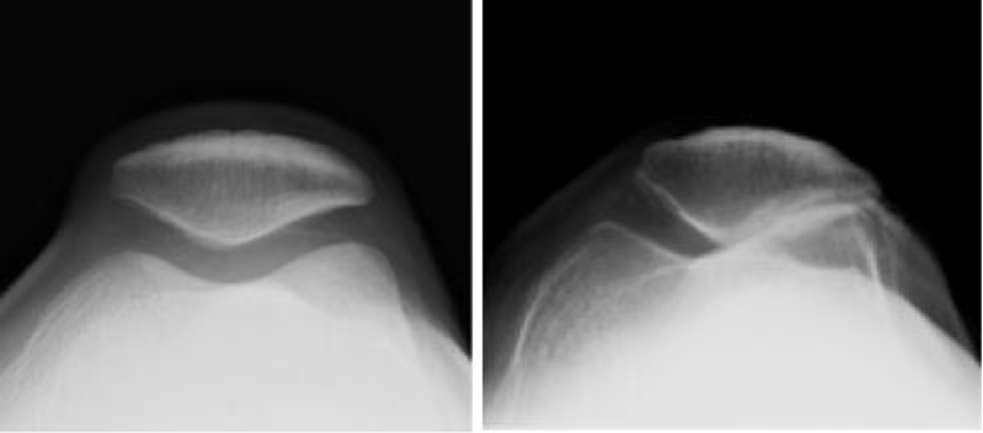
The normal (left) or advanced degenerative (right) patellofemoral joint.

In long-term friction injuries, articular cartilage wears and tears. Progressive narrowing of the PF joint space occurs. For advanced lesions, a vanished joint space and sclerosis of the subchondral bone are visible on radiographs (Fig. 3). In 1990, Iwano et al. classified PF degeneration into four stages based on radiographic findings; the advanced stage normally required surgical treatment [28,29].

Articular cartilage lesions are generally divided into grades 0–4 by Outerbridge: Grade 0, normal; grade 1, softening; grade 2, fibrillation; grade 3, fissuring; and grade 4, penetration of the bone [30]. Regardless of the cause, the treatment of articular cartilage damage is challenging. Because the cartilage is avascular, it cannot heal itself [16,23]. Once the cartilage is damaged, it may remain or worsen over time if treatment is ineffective.

Attempts have been made to repair lost articular cartilage [23,31,32]. However, despite the continuous advancement of surgical techniques, consensus regarding satisfactory outcomes is lacking. These techniques include: (1) bone marrow stimulation (subchondral drilling, microfracture, or abrasion); (2) bone marrow-derived mesenchymal stem cell implantation; (3) lipoaspirate injections; (4) platelet-rich plasma injections; (5) surgical transplantation of autologous osteochondral tissues, allogeneic osteochondral plugs, or synthetic scaffolds; and (6) regenerative transplantation [26]. Theoretically, except when PM can be completely corrected, preferentially restoring lost cartilage is generally impractical.

## Diagnosing patellar malalignment

**Fig. 4:**
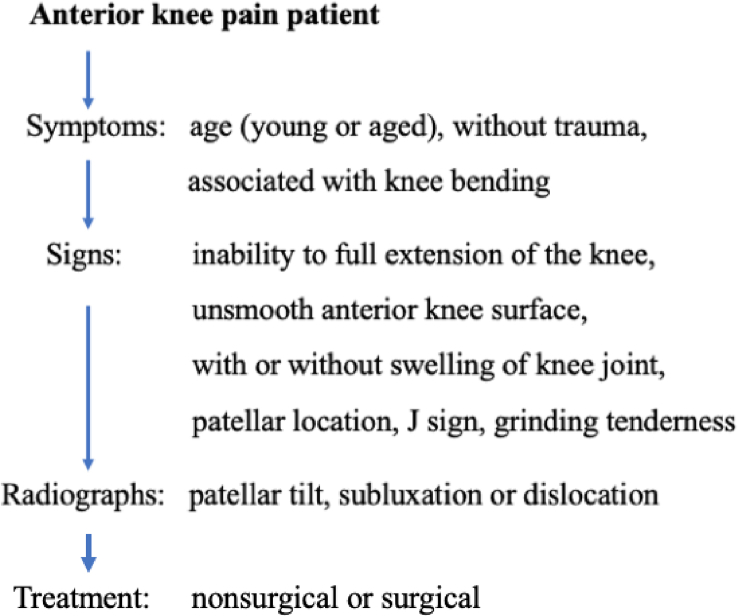
An algorithmic approach for diagnosis of isolated patellofemoral malalignment.

Knee pain is normally located behind the patella and commonly called anterior knee pain [33,34]. The reported prevalence is as high as 40% in PM patients [35]. The pain is usually vague rather than sharp and closely correlated with knee flexion (Fig. 4). In other words, patients complain of anterior knee pain while walking ascending or descending stairs, standing up from a seated position (movie sign), and squatting. In particular, knee pain while descending stairs is more evident. Such an action aggravates patellar compressive forces because of forceful knee extension to prevent giving way [36]. Knee swelling during the early degenerative stage is uncommon.

The severity of cartilage damage is closely correlated with patient age [29]. The patella possesses the thickest articular cartilage in the healthy human body, and its wear and tear are caused by long-term injury. Isolated PF lesions are not involved in TF deformities. In symptomatic PM, the knee cannot be fully extended during inspection. Knee swelling may or may not be present. The patella may be located upward (patella alta) or outward (frog-eyed patella) [37].

The grinding tenderness of the PF joint is generally obvious and laterally located. Knee flexion may induce pop shaking (J sign) [38]. In contrast, pop shaking may also occur when the knee is flexed to full extension (reverse J sign) [38]. Conceptually, radiography cannot always be used to diagnose PM. This is due to the high incidence of PM in daily life, and plain radiographs cannot detect an abnormal position of the patella in every examination [39]. Moreover, consensus is lacking on the optimal axial view for the patella: in the standing or supine position, with a relaxed or contracted quadriceps femoris, or in knee extension or flexion [40,41].

Plain radiographs with anteroposterior, lateral, and axial views are generally used for treatment planning and follow-up. Clinically, an axial view with 45° of knee bending (Merchant view) is commonly used to visualize PM [39]. Computed tomography or magnetic resonance imaging may be used to measure the tibial tuberosity-trochlear groove (TT-TG) distance, which is believed to correlate with PM [42]. Theoretically, using the trochlear groove as the point of measurement can prevent involvement of a mal-positioned patella. Measurements involving an unstable patella often have low reliability. However, the TT-TG distance can only indicate the lower portion of the Q-angle; it neglects the upper portion of the Q-angle. In the literature, the latter is reportedly more decisive than the former (2:1 effect) [43].

In 1964, Brattström arbitrarily described the Q-angle as the intersecting angle between two lines, one from the anterior superior iliac spine to the patellar center (PC) and the other from the PC to the tibial tuberosity [44]. The Q-angle has long been used to represent the action of the quadriceps femoris; however, this assertion has not been persistently validified. The main difficulty in verifying this assertion is the high incidence of PM. The Q-angle decreases with lateral patellar subluxation [45]. The Q-angle increases in the fully extended knee because the tibia screws home in the femoral condyle with external rotation [27]. Therefore, positive or negative results can be achieved. Using a goniometer to measure the Q-angle is unreliable [5,46]. In 2018, Wu et al. reported the use of a radiographic technique that avoided soft-tissue interference to accurately measure the Q-angle [29]. Accordingly, studying the correlation between the Q-angle and quadriceps femoris action may become possible despite the fact that it lacks popularity.

**Fig. 5:**
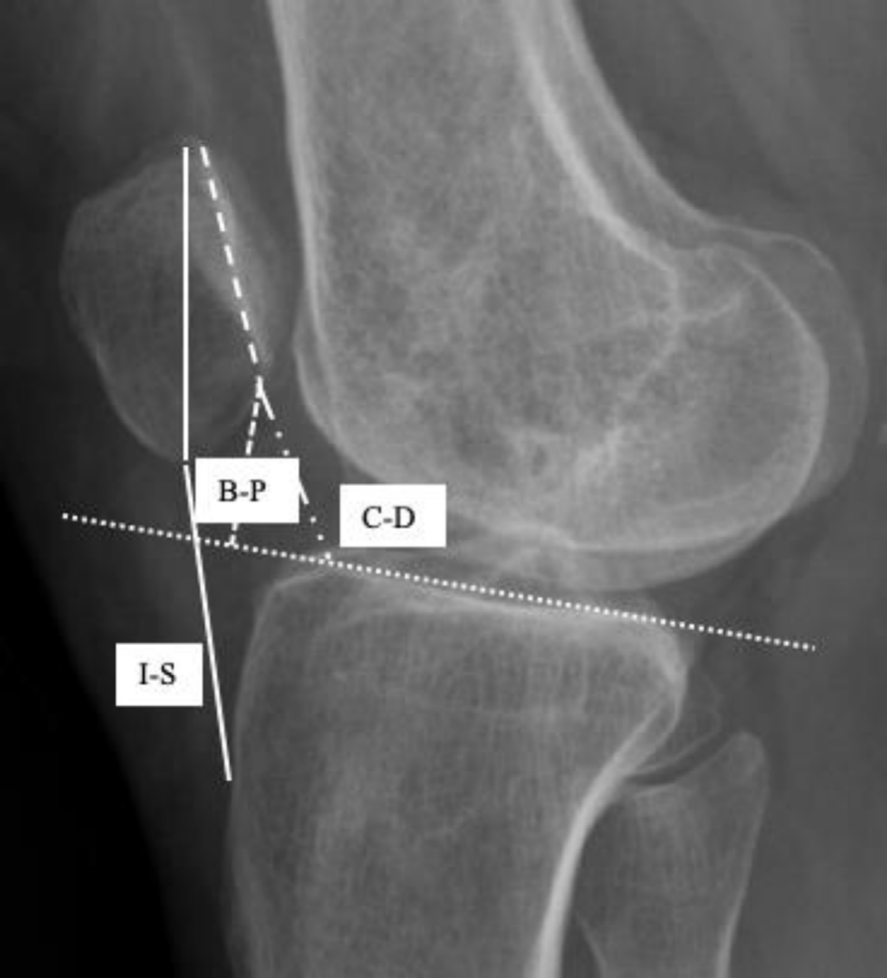
Three common methods for measuring patella alta or patella baja on the radiographs: the Insall-Salvati ratio, the Caton-Deschamps ratio or the Blackburn-Peel ratio.

To assess the patella alta or baja, lateral knee radiographs with 30° of flexion were obtained. Initially, the Insall-Salvati ratio was used to calculate the ratio of the longitudinal lengths of the patella. Because the bone contour is not always clearly visible, the Caton-Deschamps or the Blackburn-Peel ratio is used later; both can be used to calculate the articular surface between the patella and the femur (Fig. 5) [47]. The acceptable values are 0.8–1.2, 0.6–1.3, and 0.5–1.0, respectively.

## Nonsurgical treatment of patellar malalignment

**Fig. 6:**
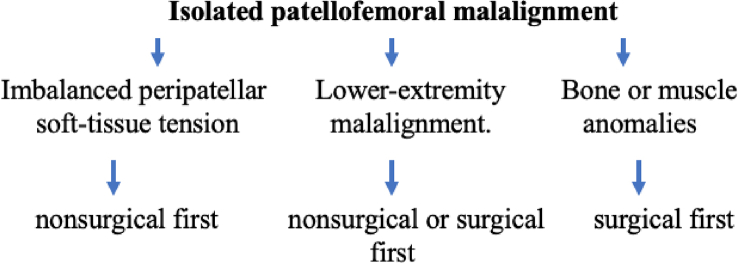
An algorithmic approach for treatment of isolated patellofemoral malalignment.

Theoretically, except for patients with surgical indications established in the literature, all PM cases should first be treated nonsurgically (Fig. 6) [48,49]. The widespread principle is to utilize techniques that can strengthen the muscle power of the VMO, gluteus, and adductor muscles [20,50].

Traditionally, conservative treatment techniques have been used in patients with low muscle power, such as squats, straight leg raises, and knee extensions with or without hip adduction. All of these techniques include either closed or open chains. However, the satisfaction rate with such approaches is always unconvincing (77%) [37]. Consequently, more effective strengthening techniques have been developed [51,52].

**Fig. 7:**
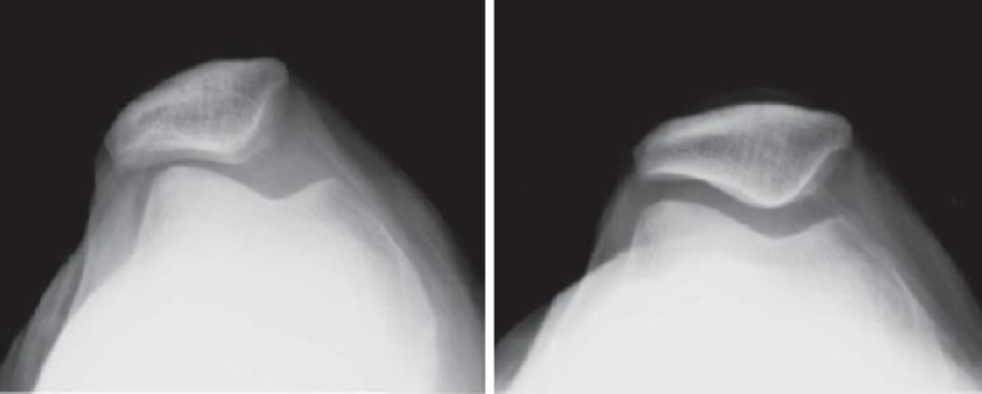
A 64-year-old woman sustained the right lateral patellar subluxation for 6 months (left). After 2-month with modified knee extension training, the patella returned to the normal position and knee pain completely subsided (right).

In 2009, the goosestep training regimen was announced [53]. In the absence of auxiliary equipment, a success rate of 72% was reported. In 2018, a modified knee extension training program was proposed [54,55]. Similarly, in the absence of auxiliary equipment, a success rate of 89% was achieved (Fig. 7). This technique uses an open chain, which has the advantage of progressively increasing knee extension strength. Goosestep training affects the muscle power of the VMO more than that of the vastus lateralis [[54], [55], [56]]. Therefore, the patella is medially pulled. The beneficial effects are attributed to three advantages of the VMO: more distal insertion on the patella, more horizontal orientation of muscle fibers, and a distally distributed main muscle mass [54,55,57].

Taping, bracing, and splinting have also been suggested by several studies, and satisfactory results have been reported [58,59]. However, in the author's opinion that long-term taping or brace use is impractical [9]. If the VMO power is not strengthened, the treatment goals with taping or brace correction will be short-term only. Both uses require a much longer time and hinder some daily activities. The reported success rate is 60–70% [58]. However, if a brace is added to VMO training, the satisfaction rate increases [59].

In cases of nonsurgical treatment failure, repeated treatments and close follow-up may be reasonable [60]. Theoretically, the success rate might increase if longer durations are awaited. Surgical treatment should be considered based on established indications published in the literature [58,59].

## Surgical treatment of patellar malalignment

Surgical techniques are chosen to restore the normal human anatomy whenever possible with maximal treatment success rates [8,61]. For patients with lower-extremity malalignment or muscle or bone anomalies, abnormal structures must be corrected as much as possible [62,63]. If this goal cannot be achieved, then supplementary techniques cannot but be used. Subsequently, the success rate decreases.

Currently, medial patellofemoral ligament (MPFL) reconstruction is increasingly used in the treatment of PM with satisfactory results [9,64]. This is considered a more reasonable treatment method for most PM cases [3,65]. However, simple MPFL reconstruction is insufficient for the treatment of PM in cases of trochlear dysplasia [66,67]. Although the restraining forces are medially restored, as long as the VMO power decreases, the nonfunctional trochlear groove cannot effectively counteract the lateral tractional forces provided by the quadriceps femoris. The function of the reconstructed MPFL gradually decreases [68]. Thus, a trochleoplasty is indicated in cases of trochlear dysplasia [24,25,69].

**Fig. 8:**
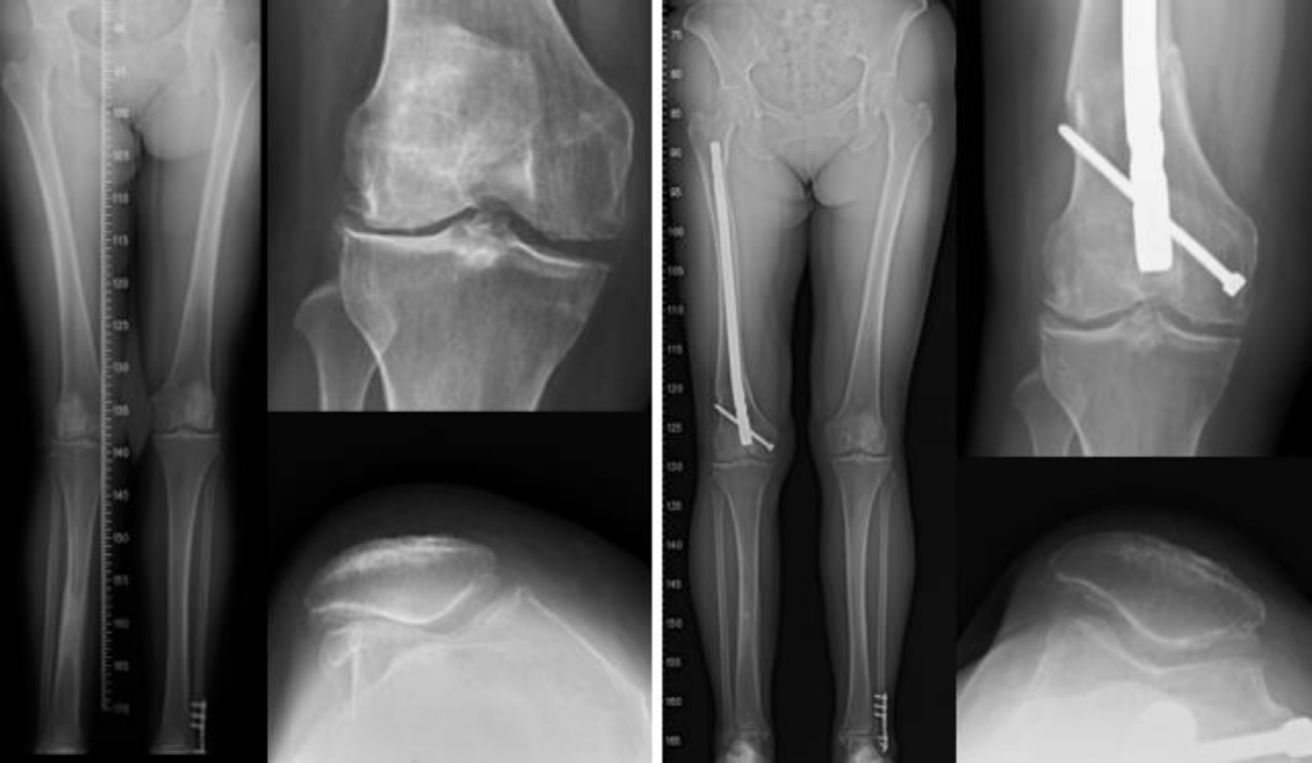
A 55-year-old woman sustained the deformed valgus knee with patellar malalignment (left). Distal femur corrective osteotomy with lateral retinacular release of the patella was performed. All anatomic abnormalities with symptoms were recovered for 2-year follow-up (right).

Patella alta can be treated by distalization of the tibial tuberosity [70,71]. Lower -extremity malalignment can be treated with a distal femoral or proximal tibial osteotomy (Fig. 8) [[72], [73], [74], [75]]. The Q-angle can be decreased by a tibial tuberosity osteotomy of Fulkerson [76,77]. In general, single or combined surgical techniques yield improved results.

**Fig. 9:**
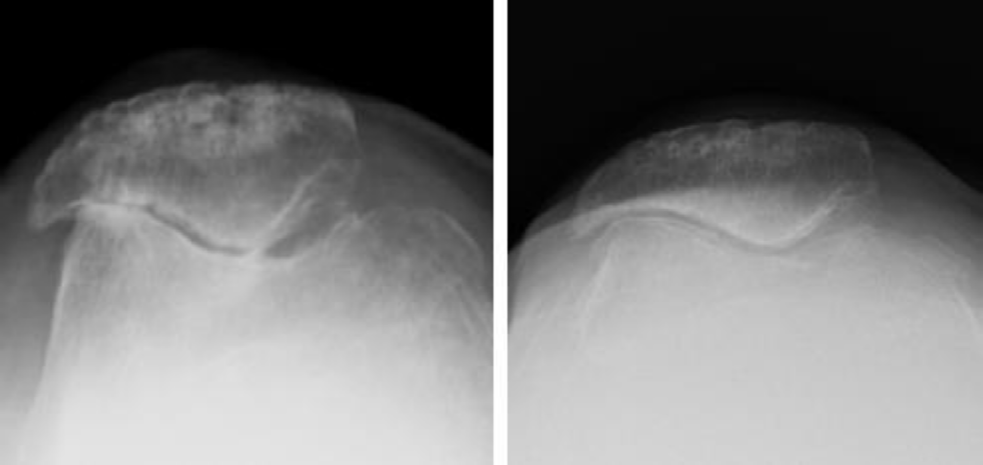
A 58-year-old woman sustained the right advanced isolated patellofemoral osteoarthritis with patellar malalignment (left). The combined lesions were treated with lateral retinacular release and drilling chondroplasty. Postoperatively, knee extension training was advised whenever necessary. All symptoms subsided with 5-year follow-up (right).

Because the Q-angle and the ITB place outward forces on the patella during knee flexion-extension, simply performing a lateral retinaculum release on the patella normally has limited efficacy [63,68,78]. In contrast, combined lateral retinacular release with VMO strengthening exercises improve the results (Fig. 9) [29,59].

An isolated PF arthroplasty or total knee arthroplasty may be indicated [79,80]. Clinicians should consider patient age, activity level, and surgical risks. Modified knee activity is normally tolerable for most patients in daily life [81].

## Conclusions

Knee pain caused by PM is common and should not be neglected. PM is largely caused by imbalanced peripatellar soft-tissue tension, and nonsurgical treatment is normally effective. Knee pain is associated with bending the knee (e.g., ascending or descending stairs, standing from a seated position, or squatting). Although PM can occur in any age group, it should be preferentially considered in young individuals with non-traumatic knee pain.

## Funding

There is no funding in supporting this study.

## Conflict of interests

The author declares that there are no conflicts of interest in this study.
